# Insights into cisplatin-induced neurotoxicity and mitochondrial dysfunction in *Caenorhabditis elegans*

**DOI:** 10.1242/dmm.049161

**Published:** 2022-03-31

**Authors:** Carmen Martínez-Fernández, Milana Bergamino, Alfonso Schiavi, David Brena, Natascia Ventura, Sebastian Honnen, Alberto Villanueva, Ernest Nadal, Julián Cerón

**Affiliations:** 1 Modeling Human Diseases in C. elegans Group; Genes, Diseases, and Therapies Program, Institut d'Investigació Biomèdica de Bellvitge - IDIBELL, L'Hospitalet de Llobregat, 08908 Barcelona, Spain; 2 Department of Medical Oncology, Breast Cancer Unit, Catalan Institute of Oncology, Hospital Duran i Reynals, Avda Gran via, 199-203, L'Hospitalet, 08908 Barcelona, Spain; 3 Institute of Clinical Chemistry and Laboratory Diagnostic, Medical Faculty, Heinrich Heine University, and the Leibniz Research Institute for Environmental Medicine, 40225 Düsseldorf, Germany; 4 Institute of Toxicology, Medical Faculty, Heinrich Heine University, Universitätsstraße 1, D-40225 Düsseldorf, Germany; 5 Group of Chemoresistance and Predictive Factors, Subprogram Against Cancer Therapeutic Resistance, Catalan Institute of Oncology, Oncobell Program, Bellvitge Biomedical Research Institute (IDIBELL), L'Hospitalet del Llobregat, 08908 Barcelona, Spain; 6 Medical Oncology Department, Catalan Institute of Oncology, L'Hospitalet, 08908 Barcelona, Spain

**Keywords:** *C. elegans*, CRISPR-Cas9, Cisplatin, Glucose, Mitochondria, Neurotoxicity

## Abstract

Cisplatin is the most common drug in first-line chemotherapy against solid tumors. We and others have previously used the nematode *Caenorhabditis elegans* to identify genetic factors influencing the sensitivity and resistance to cisplatin. In this study, we used *C. elegans* to explore cisplatin effects on mitochondrial functions and investigate cisplatin-induced neurotoxicity through a high-resolution system for evaluating locomotion. First, we report that a high-glucose diet sensitizes *C. elegans* to cisplatin at the physiological level and that mitochondrial CED-13 protects the cell from cisplatin-induced oxidative stress. Additionally, by assessing mitochondrial function with a Seahorse XFe96 Analyzer, we observed a detrimental effect of cisplatin and glucose on mitochondrial respiration. Second, because catechol-*O*-methyltransferases (involved in dopamine degradation) are upregulated upon cisplatin exposure, we studied the protective role of dopamine against cisplatin-induced neurotoxicity. Using a Tierpsy Tracker system for measuring neurotoxicity, we showed that abnormal displacements and body postures in *cat-2* mutants, which have dopamine synthesis disrupted, can be rescued by adding dopamine. Then, we demonstrated that dopamine treatment protects against the dose-dependent neurotoxicity caused by cisplatin.

## Abstract (precis)

**Summary:** Using *Caenorhabditis elegans* to explore neuronal and metabolic factors influencing cisplatin toxicity revealed that dopamine treatment protects against the dose-dependent neurotoxicity caused by cisplatin.

## INTRODUCTION

Cis-diammine dichloride platinum (CDDP), also known as cisplatin, is one of the most used platinum derivatives and the one with the highest therapeutic efficacy in a series of solid tumors, including testicular, ovarian, cervical, bladder, head and neck, and lung cancer (Amable, 2016; Dasari and Bernard Tchounwou, 2014). The most remarkable example is testicular cancer, for which cisplatin provides a cure for more than 80% of patients (Gonzalez-Exposito et al., 2016). Unfortunately, despite its effectiveness, oncologists need to deal with three main inconveniences associated with its use, which ultimately result in therapy failure and increased mortality: (1) the tumor-acquired resistance (Amable, 2016), (2) the intrinsic resistance of many patients and (3) the toxic side effects (Kelland, 2007). Therefore, the identification of factors predicting dosage sensitivity to therapy and finding of novel resensitizing therapeutic approaches are essential for effective treatment with cisplatin.

Besides genetic background, the metabolic profile also influences cancer progression and outcome, and response to chemotherapy. Metabolic disorders, such as type 2 diabetes mellitus, negatively affect the clinical outcome of cancer patients (Barone et al., 2008; Harding et al., 2015; Pearson-Stuttard et al., 2015). Recently, it has been suggested that fasting plasma glucose (FPG) is a predictor of survival in non-small cell lung cancer (NSCLC) patients treated with concurrent chemoradiotherapy because higher FPG correlates with a lower overall survival rate (Bergamino et al., 2019). Therefore, although a good metabolic control would improve cancer outcomes, there are conflicting data supporting the efficiency of antidiabetic compounds in reducing cancer mortality (Lin et al., 2015; Ranc et al., 2014; Shlomai et al., 2016). The addition of metformin to chemoradiotherapy in non-diabetic patients with locally advanced NSCLC did not improve overall survival (Skinner et al., 2021; Tsakiridis et al., 2021).

The unspecific mode of action of cisplatin works as a double-edged sword, affecting both tumoral and normal cells. CDDP undergoes aquation when entering the cell, becoming more reactive to interact with a broad range of cellular targets, both in the nucleus and cytoplasm (Kelland, 2007). Among the cytotoxic consequences of cisplatin, the formation of DNA-cisplatin adducts and the production of reactive oxygen species (ROS), which lead to homeostasis imbalance and ultimately provoke apoptosis, are considered the canonical mechanisms of action (Wang and Lippard, 2005). Thus, the need to reduce toxic side effects implies a dose-limiting outcome in cisplatin-based therapies.

The most common side effect is ototoxicity, affecting more than 60% of pediatric cancer patients, followed by peripheral nervous system toxicity and nephrotoxicity. This particular toxicity leads to partial or even complete hearing loss, compromising the language and cognitive development of these patients (Ross et al., 2009). Interestingly, catechol-*O*-methyltransferase (*COMT*), acylphosphatase 2 (*ACYP2*), and thiopurine methyltransferase (*TPMT*) genetic variants have been related to ototoxicity (Ross et al., 2009). However, further reports show contradictory data (Thiesen et al., 2017), and the mechanisms undergoing this common pathophysiology remain obscure.

*Caenorhabditis elegans* has been successfully used as an experimental organism since the 1970s, providing several advantages over other models. This nematode is a well-established system to investigate neuromodulatory pathways *in vivo* (Van Damme et al., 2021) and the effects of genotoxic drugs in a pluricellular context (Honnen, 2017; Kaletta and Hengartner, 2006). Particularly, we and others have recently demonstrated the value of *C. elegans* to explore genetic, cellular and molecular factors involved in cisplatin response (Hemmingsson et al., 2010; García-Rodríguez et al., 2018; Wellenberg et al., 2021). Here, exploiting our previous methodologies, along with CRISPR-Cas9 technology and semi-automated platforms (Seahorse XFe96 Analyzer and Tierpsy Tracker), we further investigated the effects of cisplatin-based therapies. We observed that a glucose-enriched diet negatively impacts cisplatin response, particularly affecting mitochondrial function. Moreover, we demonstrate the protective role of the BH3-only protein CED-13 against cisplatin-induced mitochondrial ROS (mtROS). We also found that dopamine (DA) protects against the effects of cisplatin-induced neurotoxicity on animal locomotion.

## RESULTS

### High-glucose diet influences the animal response to cisplatin

**Fig. 1.**
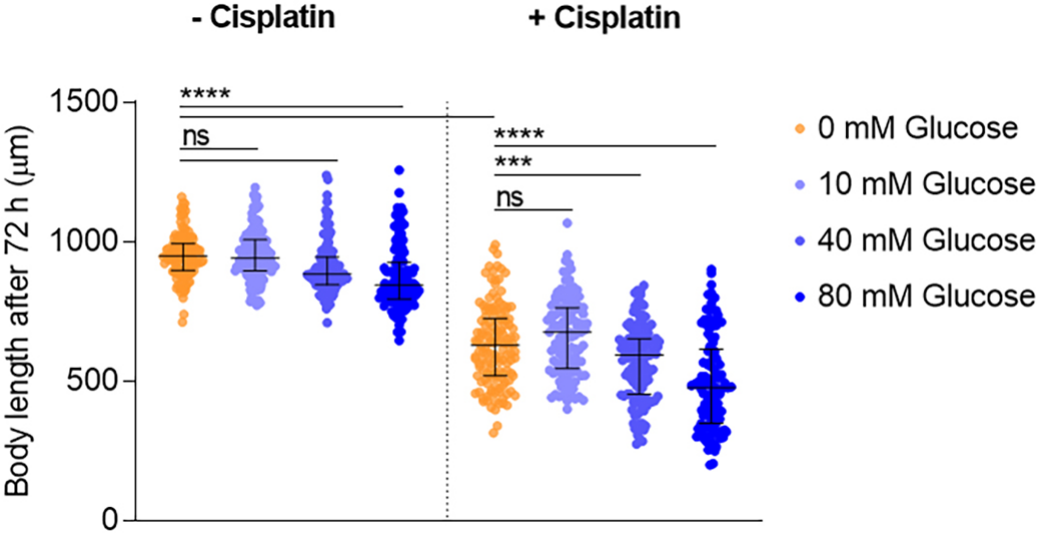
**Glucose supplementation enhances cisplatin’s effect on *C. elegans* body length.** The graph shows body length at 72 h post-seeding in L1 animals at 20°C, fed with different glucose concentrations, and exposed or not to 60 µg/ml cisplatin. Bars indicate the median and interquartile range, and dots indicate body length values of individual animals (50 animals per condition in each experiment) in three independent experiments. ns, non-significant; ***P<0.001, ****P<0.0001. Statistical analysis was performed with ordinary one-way ANOVA (Holm–Sidak's test).

In a previous study, we described a dose-response effect of cisplatin on *C. elegans* body length during postembryonic development (García-Rodríguez et al., 2018). In the same report, we identified the insulin/IGF-1 signaling (IIS) pathway as a critical regulator in the animal response to cisplatin, suggesting the relevance of metabolism in cisplatin chemosensitivity. To investigate whether hyperglycemic conditions impact cisplatin response in the nematode, we assessed the effect of a high-glucose diet on animal body length during larval development under our standard cisplatin conditions (60 µg/ml). Animals exposed to this concentration of cisplatin from L1 larval stage become sterile, present excess of apoptosis and do not reach adulthood. However, body length was a reliable indicator of cisplatin sensitivity (García-Rodríguez et al., 2018). We performed a dose-response assay including glucose concentrations in *C. elegans* whole-body extract of 10-15 mM, resembling the glucose levels of diabetic patients under poor glucose control (Schlotterer et al., 2009). Our data showed that high-glucose diet does not cause a major effect on *C. elegans* development, but, when co-administered with cisplatin, high glucose concentrations (40 mM and 80 mM) sensitize the animals to cisplatin (Fig. 1).

### The combination of paraquat (PQ) and cisplatin causes an additive adverse effect

**Fig. 2.**
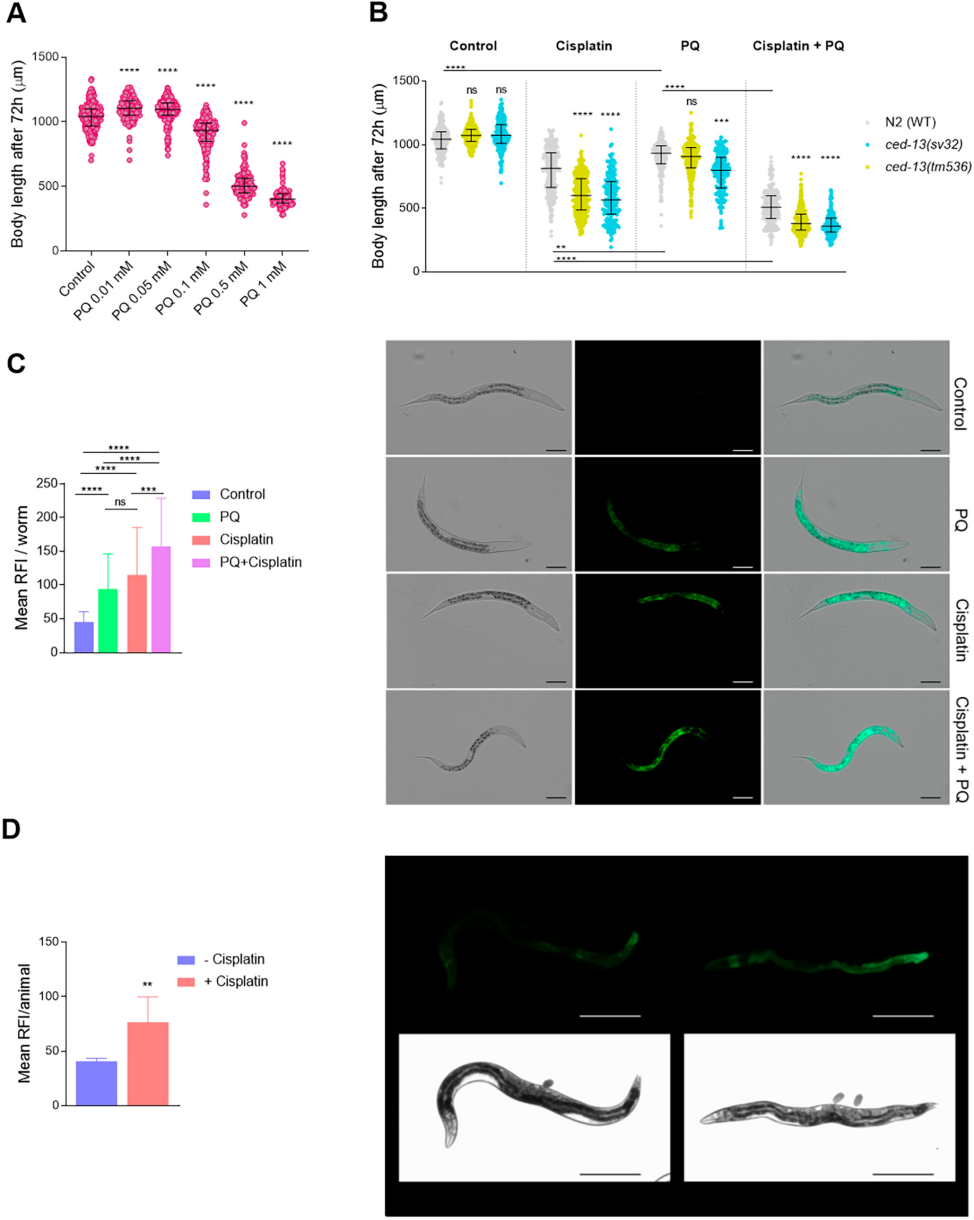
**Cisplatin potentiates the effect of the pro-oxidant paraquat (PQ) and activates mitochondrial damage response pathways.** (A) Dose-response curve showing PQ effect on wild-type (WT) animals' body length. Statistical analysis was performed with one-way ANOVA (Kruskal–Wallis and Dunn's tests). ****P<0.0001. (B) Additive effect of 60 µg/ml cisplatin and 0.1 mM PQ in WT and *ced-13* mutants (*sv32* and *tm536*). Statistical analysis was performed with one-way ANOVA (Kruskal–Wallis and Dunn's tests). ns, non-significant; ***P<0.001, ****P<0.0001, compared to WT in the same drug condition. Three independent experiments were performed, analyzing a total number of 150 animals per condition. (C) *gst-4::GFP* relative fluorescence intensity (RFI) in control, 60 µg/ml cisplatin, 0.1 mM PQ and combination of cisplatin and PQ-treated animals for 24 h from L1 stage. The experiment was performed three times, measuring 20 animals per condition. Statistical analysis was performed with one-way ANOVA (Kruskal–Wallis and Dunn's tests). ns, non-significant; ***P<0.001, ****P<0.0001. Representative differential interference contrast (DIC) and fluorescence images of animals expressing *gst-4::GFP* are shown on the right. Scale bars: 50 µm. (D) *hsp-6::GFP* relative fluorescence intensity (RFI) for control and 100 µg/ml cisplatin cisplatin-treated animals. Bars represent the mean of three independent experiments (10-15 animals per condition were analyzed in each experiment) and lines the s.d. Statistical analysis was performed with unpaired, two-tailed Student's *t*-test. **P<0.01. Representative DIC and fluorescence images of animals expressing *hsp-6::GFP* under control and cisplatin conditions are shown on the right. Scale bars: 250 µm.

High-glucose levels alter mitochondrial properties and functions in *C. elegans* (Alcántar-Fernández et al., 2019). We investigated whether glucose enhancing the effect of cisplatin on body length could occur through oxidative stress induction and ROS generation. Thus, we tested whether cisplatin displays a cumulative effect with the oxidant PQ, a potent mtROS generator. First, we assessed the dose-dependent impact of PQ on *C. elegans* development (Fig. 2A). A mild toxic PQ dose (0.1 mM), high enough to provoke a significant reduction in body length but allowing the study of its impact in combination with cisplatin, was used for subsequent experiments. Interestingly, PQ (0.1 mM) produced an enhancement of cisplatin’s (60 µg/ml) effect on body length (Fig. 2B), similar to that observed with high-glucose diet (Fig. 1). Because cisplatin causes an increase in total ROS levels in adult *C. elegans* (Raj et al., 2021), we asked whether PQ potentiates the induction of ROS in cisplatin-treated animals. Consequently, using an indicator of oxidative stress level, *gst-4::GFP* (Tawe et al., 1998), we observed an additive effect of PQ (0.1 mM) and cisplatin (60 µg/ml) on *gst-4* levels, which are indicative of ROS production (Fig. 2C). These results support that toxicity caused by cisplatin in combination with glucose or PQ occurs by oxidative stress induction.

The *C. elegans* BH3-only protein CED-13 promotes cell survival in response to mtROS, instead of inducing apoptosis (Yee et al., 2014). Moreover, as we have previously described, CED-13 protects *C. elegans* against cisplatin-induced toxicity (García-Rodríguez et al., 2018) (Fig. 2B). To confirm that cisplatin and PQ additive toxicity occurs through the production of ROS, we used two different *ced-13* mutant alleles, *sv32* and *tm536*, harboring 1304 bp and 523 bp deletions, respectively. We use two alleles because *sv32* deletion also affects a neighboring gene and *tm536* is a ‘cleaner’ deletion, removing the start codon. Interestingly, we observed that CED-13 also protects against cisplatin and PQ additive toxic effects (Fig. 2B).

Given the central role of mitochondria in cellular metabolism, we decided to evaluate whether this organelle was one of the cisplatin targets by assessing the activation of the mitochondrial unfolded protein response (UPR^mt^) pathway. The UPR^mt^ pathway is induced in response to misfolded or unassembled proteins within the mitochondria or when mitochondrial respiratory complexes are imbalanced. To check the UPR^mt^ activation, we quantified the expression of HSP-6, a chaperone commonly used to rate UPR^mt^ pathway activation (Jovaisaite et al., 2014). L4/YA animals treated with 100 µg/ml cisplatin exhibited increased expression of the *hsp-6::GFP* reporter (Fig. 2D). This observation suggests that cisplatin may have a detrimental effect on mitochondrial functions.

### Impact of cisplatin and glucose on mitochondrial respiration

**Fig. 3.**
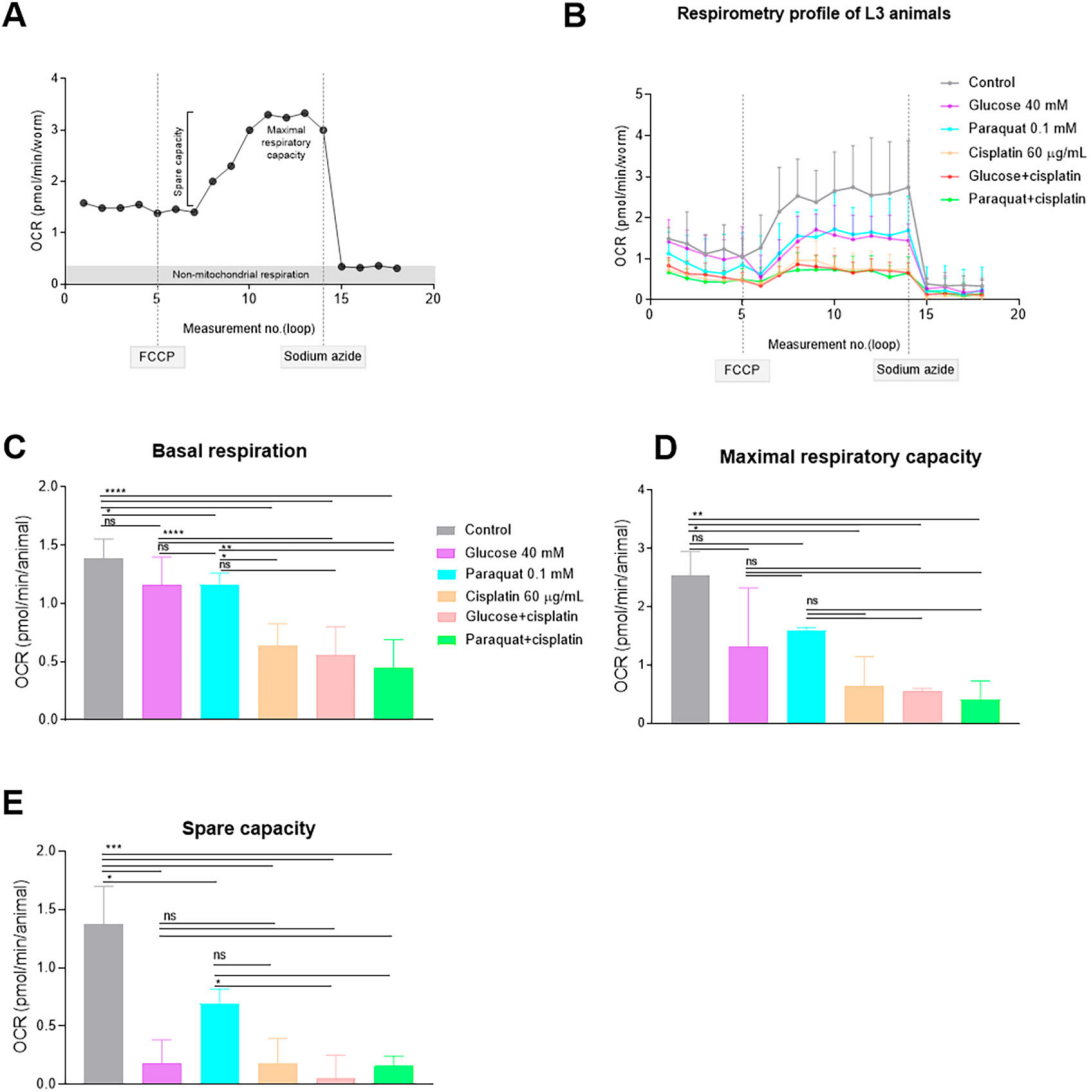
***C. elegans* respirometry evaluation.** (A) Typical oxygen consumption rate (OCR) respirometry profile in adult *C. elegans* animals. Based on Koopman et al. (2016). This image is not published under the terms of the CC-BY license of this article. For permission to reuse, please see Koopman et al. (2016). Before drug injections, the respirometer informs about basal respiration. Then, FCCP injection disrupts the mitochondrial membrane potential and ATP synthesis while still allowing proton pumping, electron transport and oxygen consumption. Thus, FCCP enables the measurement of maximal respiratory capacity. The extraction of the basal respiration from the maximal respiratory capacity results in the spare respiratory capacity, a value indicating the organism’s ability to respond to increasing energy demands. Finally, injection of sodium azide blocks both cytochrome c oxidase (complex IV) and the ATP synthase (complex V), thereby shutting down the whole electron transport chain and allowing the distinguishing of non-mitochondrial oxygen consuming processes. (B) OCR profile of *C. elegans* L3 stage larvae in control and treated conditions. Connected points represent the median of the measures of each condition in a given loop and lines represent s.d. Dashed lines indicate FCCP and sodium azide injections. (C-E) Median and interquartile range are represented by bars and error bars, respectively, for basal respiration (C), maximal respiratory capacity (D) and spare capacity (E). This experiment was performed in triplicates, including eight biological replicates for each condition (a total of 160 animals per treatment). One-way ANOVA (Holm-Sidak's and Dunn's tests) was used to compare statistical differences between groups. ns, non-significant; *P<0.1, **P<0.01, ***P<0.001, ****P<0.0001. Data were analyzed using Agilent Seahorse XFe96 Analyzer, Seahorse Wave Desktop software and GraphPad Prism 8.0.

The disruption of the stoichiometric balance between components of mitochondrial respiratory complexes (OXPHOS complexes I, III, IV and V) is one of the signals triggering UPR^mt^ (Jovaisaite et al., 2014). To investigate the impact of cisplatin and high-glucose levels on the activity of OXPHOS complexes, we assessed the mitochondrial respiration of L3 animals using a Seahorse XFe96 Analyzer, including PQ-treated animals as a control group. The Seahorse XFe96 Analyzer facilitates the measurement of the oxygen consumption rate (OCR), a measure of mitochondrial function and energy production rate, before and after the sequential injection of two mitochondrial complex inhibitors – carbonyl cyanide-4-(trifluoromethoxy) phenylhydrazone (FCCP) and sodium azide (Koopman et al., 2001) – thus allowing extraction of the basal respiration, maximal respiratory capacity and spare respiratory capacity values (Fig. 3A). We measured the OCR in L3 larvae after being exposed for 24 h to the corresponding treatment. As expected, cisplatin dramatically affected the basal respiration (Fig. 3B,C), maximal respiratory capacity (Fig. 3B,D) and spare capacity (Fig. 3E). Such parameters were also affected by other mtROS producers such as glucose and PQ, indicating their detrimental effects at mitochondrial level.

### COMT genes are involved in DA-dependent processes

One of the intriguing genes that showed upregulation upon cisplatin exposure in our previous publication was *comt-4*, which encodes a catechol-*O*-methyltransferase related to the degradation of catecholamines, including DA (García-Rodríguez et al., 2018). Because genetic variants of the human *COMT* gene have been linked to ototoxicity in cisplatin-treated children (Ross et al., 2009; Thiesen et al., 2017), we decided to investigate in our model the role of COMT genes in cisplatin-induced neurotoxicity. The *C. elegans* COMT family has five members (*comt-1* to *comt-5*), all containing the catechol-*O*-methyltransferase domain. In humans, there are two genes involved in catechol-O-methyltransferase activity: *COMTD1* and *COMT*. The human *COMT* gene encodes two similar isoforms, S-COMT and M-COMT, with 100% sequence similarity but with different subcellular localization. BLASTP analysis, using S-COMT as a reference sequence, shows high sequence similarity with *C. elegans* COMT members, particularly at the C-terminal (Fig. S1, Table S1). Interestingly, despite COMTD1 being the most closely related in terms of sequence similarity to *C. elegans* COMTs, most of the binding sites for S-COMT ligands are conserved or partially conserved along all the *C. elegans* COMTs, suggesting the conservation of functional roles. Among them, COMT-4 possesses the highest percentage of amino acid identity per query cover (Table S1) with human S-COMT (Rodríguez-Ramos et al., 2017). A *comt-4* endogenous transcriptional reporter generated by Nested CRISPR (Vicencio et al., 2019) displayed expression in neuronal cells (Fig. S2), as expected from its functional role and the transcriptomic data from GExplore (Cao et al., 2017; Hutter and Suh, 2016).

Because the five *C. elegans* COMT family members could present certain functional redundancy, besides *comt-4*, we also investigated *comt-3* and *comt-5* because of their higher expression at postembryonic stages compared to *comt-1* and *comt-2* (Hutter and Suh, 2016). Thus, we generated deletion alleles for the three genes by CRISPR-Cas9 [*comt-3(cer130)*, *comt-4(cer126)* and *comt-5(cer128)*] (Fig. S3).

First, we examined body length, which is negatively regulated by DA (Nagashima et al., 2016), in the three COMT mutants. As an experimental control, we used a null allele for *cat-2*, a gene encoding a tyrosine hydroxylase required for DA synthesis. *cat-2(n4547)* mutants present reduced levels of DA (Smith et al., 2019), thus affecting DA-dependent behavioral and morphological effects (Omura et al., 2012; Smith et al., 2019). Body lengths of synchronized animals were measured at 96 h post-seeding. As expected, *cat-2(n4547)* DA-defective animals were larger than wild-type (WT) animals. In contrast, *comt-5(cer126)* animals, and a triple mutant strain harboring *comt-3*, *comt-4* and *comt-5* deletion alleles, were shorter than WT (Fig. S4). This result suggests that *comt-5* has a greater effect on DA catabolism than *comt-3* and *comt-4*. Nonetheless, we cannot discard an impact of other COMT genes and functional redundancies on distinct DA-regulated processes.

### COMT mutants show behavioral phenotypes even in the absence of cisplatin

**Fig. 4.**
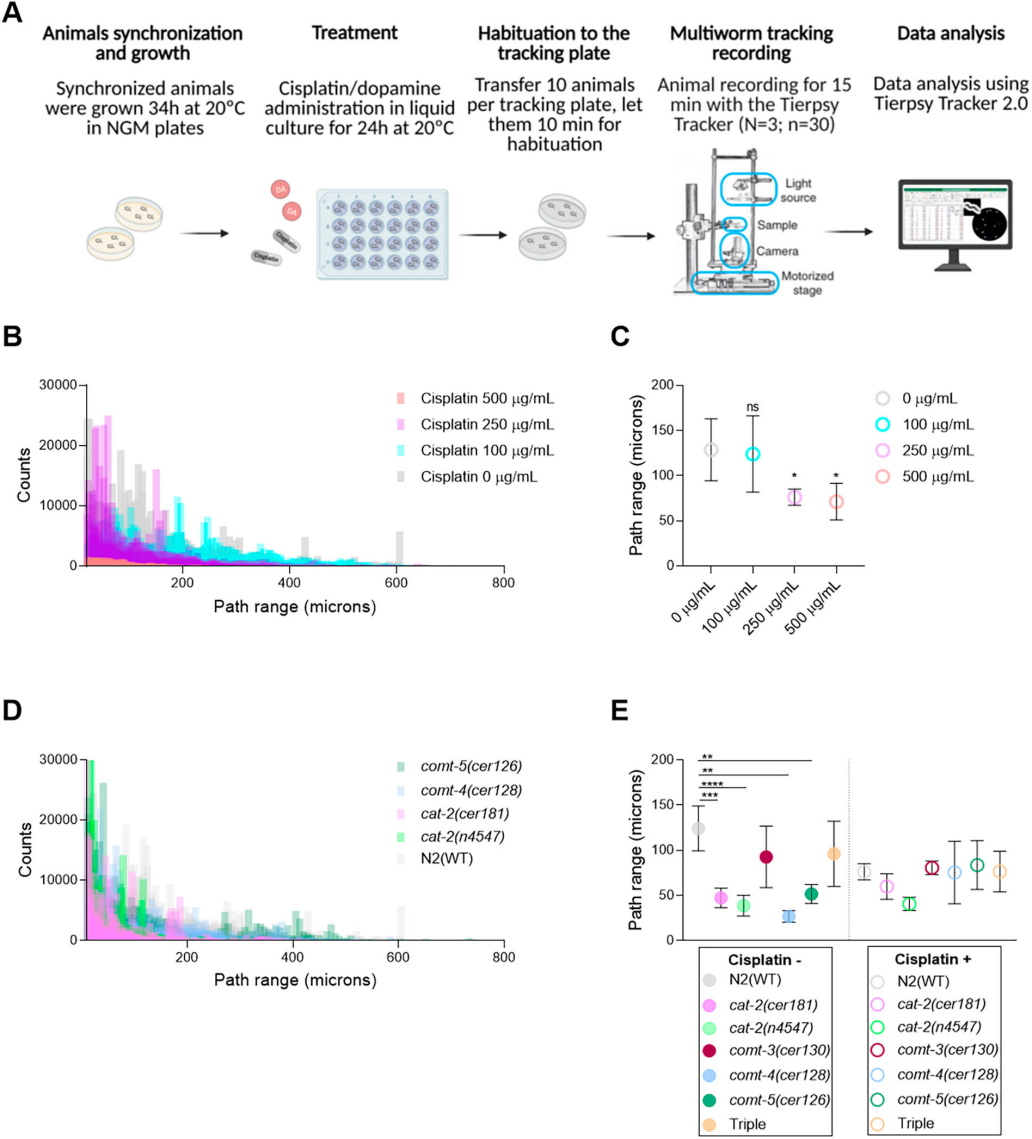
**Neurotoxic evaluation using the Tierpsy Tracker in WT and dopamine (DA) signaling-related mutants.** (A) Schematic representation of the experimental flow followed to evaluate neuronal functions under control and treated (cisplatin and DA) conditions. Created with Biorender.com. (B) Histograms show the dose-dependent effect of cisplatin on path range in WT animals. (C) Circles represent the mean path range of animals exposed to cisplatin; lines represent s.d. (D) Histograms represent the path range profile of WT, and *cat-2*, *comt-4* and *comt-5* mutants. (E) Circles represent the mean path range of animals exposed or not to 250 µg/ml cisplatin. Lines represent s.d. of two independent experiments. 30 animals per condition were evaluated in each experiment. Statistical analysis was performed with one-way ANOVA (Kruskal­­–Wallis and Dunn's tests). ns, non-significant; *P<0.1, **P<0.01, ***P<0.001, ****P<0.0001.

To study cisplatin-induced neurotoxicity in *C. elegans*, we evaluated different DA-dependent phenotypes in control and cisplatin conditions. However, none of these standard assays clearly showed differences between WT and COMT mutants. Thus, we established a methodology to automatically track animals using the Tierpsy Tracker (Javer et al., 2018a) (Fig. 4A). This system combines the throughput of multi-worm tracking with the resolution of single worm movements, allowing the extraction of detailed phenotypic fingerprints from a population (Stephens et al., 2008).

We determined cisplatin conditions leading to an altered behavioral phenotype in adult WT animals. L4 animals were treated with distinct cisplatin concentrations (100, 250 and 500 μg/ml) for 24 h. Then, nematodes were transferred to tracking plates and recorded for 15 min. Finally, we processed data files using Tierpsy Tracker 2.0 software (Javer et al., 2018b) (Fig. 4A). Cisplatin produced a dose-dependent decrease in the overall path range, defined as the distance of the animal's midbody from the path centroid measured in microns (Fig. 4B,C; Fig. S5 and Movie 1). We used 250 μg/ml as the standard concentration of cisplatin on plates because it was the highest possible dose that did not affect viability. Moreover, we did not observe changes in dopaminergic neurons integrity or cell loss (Fig. S6) when animals were exposed to 250 µg/ml cisplatin for 24 h from L4/YA stage.

Thus, we used the tracker system to study the impact of cisplatin (250 μg/ml) on the locomotor activity of distinct mutant backgrounds. As a control, we included two *cat-2* deletion alleles, *cer181* and *n4547*. By CRISPR-Cas9, we generated the allele *cer181* because the *n4547* allele affects the untranslated region of an additional locus (*pqn-85*). These *cat-2* mutants were expected to show impaired locomotion compared to WT animals due to hampered DA signaling (Omura et al., 2012; Smith et al., 2019). We subjected both *cat-2* mutants along with COMT mutants [*comt-3(cer130)*, *comt-4(cer126)*, *comt-5(cer128)* and triple mutant] and WT animals to path range evaluation in control and cisplatin conditions. Significant reduction in the traveled distances compared to WT was evident in untreated conditions, not only for *cat-2* mutants (Movie 2) but also for *comt-4* and *comt-5* deletion alleles (Fig. 4D,E; Fig. S5). Path ranges were not further altered in any mutant strain under cisplatin exposure (Fig. 4E). Thus, although we expected WT locomotion in COMT null mutants, they displayed a behavioral phenotype too strong in control conditions to study their sensitivity to cisplatin in our experimental conditions.

### DA protects against cisplatin-induced neurotoxicity

**Fig. 5.**
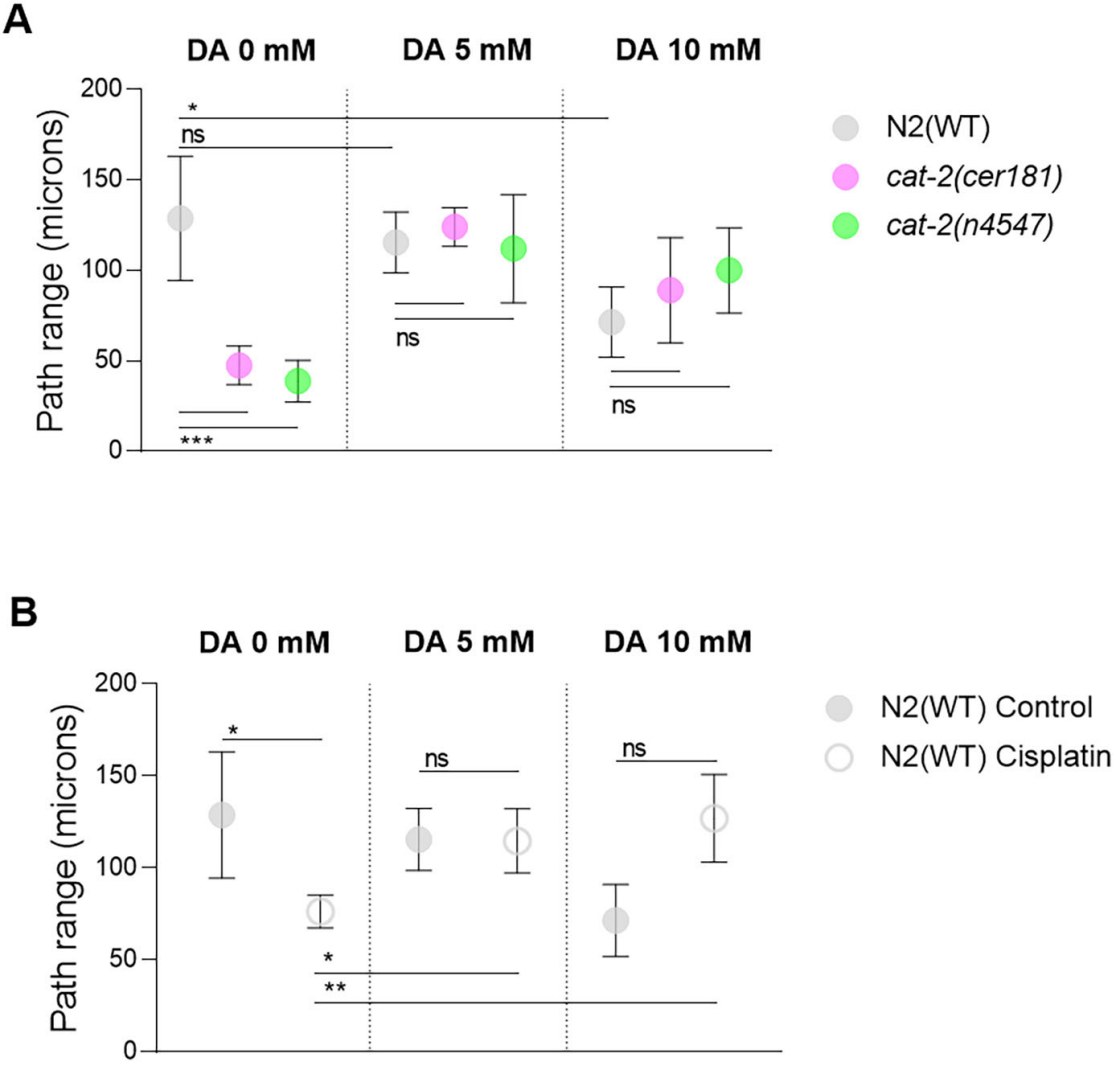
**DA influences path range and protects from cisplatin-induced neurotoxicity.** (A,B) DA rescues the behavioral defects of low-DA mutants (A) and 250 µg/ml cisplatin-exposed animals (B). Circles represent the mean path range of control animals or those exposed to cisplatin. Lines represent s.d. DA concentration is indicated at the top of the graphs. This experiment was performed three times. Control N2 (WT) samples are common for A and B. Three biological replicates were evaluated in each experiment, with a total of 30 animals per condition. Statistical analysis was performed with one-way ANOVA (Kruskal–Wallis and Dunn's tests). ns, non-significant; *P<0.1, **P<0.01.

DA-deficient mutants, *cat-2(cer181)* and *cat-2(n4547)*, traveled shorter distances than animals with normal levels of DA (Fig. 4D,E; Fig. S5 and Movie 2). To study the neuroprotective effect of DA in cisplatin-treated animals, we first tested the efficacy of exogenous DA (5 Mm and 10 Mm) in live animals by rescuing the locomotor phenotypes of *cat-2* mutants. Low DA supplementation (5 mM) was enough to increase *cat-2* mutants path ranges up to WT values. Interestingly, we observed a detrimental effect of DA at 10 mM in the WT background (Fig. 5A), further supporting that proper DA levels are essential to maintain normal displacements. Given the influence of DA on animal displacement, we asked whether exogenous supplementation of this compound could protect against the neurotoxicity caused by cisplatin. Strikingly, we noticed that 5 mM and 10 mM DA rescued the cisplatin-altered path range to WT standards, with 10 mM being the most effective dosage (Fig. 5B; Movie 3).

The Tierpsy Tracker not only reports locomotor features (path range) but also body postures adopted by nematodes (Javer et al., 2018b). The posture of the worm can be reconstructed as a summation of eigenworms or eigenprojections. We considered six eigenprojections (*α*_1_-*α*_6_), which can almost completely describe the natural worm posture by measuring the worm curvature (Fig. S7A). We observed *cat-2* mutants with abnormal body postures (Fig. S7B) and an interesting synergistic effect on altering body posture between *cat-2(cer181)* mutants and cisplatin (Fig. S7C). We noticed that 5 mM and 10 mM exogenous DA could also rescue altered body postures of *cat-2(n4547)*, and *cat-2(cer181)* plus cisplatin, to WT values (Fig. S7C). Altogether, our data indicate that DA regulates locomotion and body postures, and protects against cisplatin-induced neurotoxicity in *C. elegans*.

## DISCUSSION

Our previous findings revealed that the conserved IIS pathway is involved in cisplatin response by activating its main transcription factor DAF-16/FOXO (García-Rodríguez et al., 2018). The IIS pathway, through diet, regulates a broad variety of processes, such as stress resistance, innate immunity and metabolic adaptation in animals (Singh and Aballay, 2009; Yang and Hung, 2009; Murphy and Hu, 2013). Although high-glucose diets cause a myriad of phenotypes (such as reproductive alterations and aging) and metabolic changes (such as lipid composition and fat accumulation) (Alcántar-Fernández et al., 2019; Mejia-Martinez et al., 2017), we observed an additive impact on animal development between cisplatin and moderate and high glucose levels (40 mM and 80 mM, respectively). Thus, metabolism influences cisplatin response and consequently could be modulated for more efficient chemotherapy. Adaptive metabolic responses to oxidative agents, including a reduction in the core biological processes, have been reported in distinct animal models (Shore et al., 2012; Ventura et al., 2009).

The mitochondrial BH3-only CED-13 protein protects against mtROS (Yee et al., 2014), and, consistently, we demonstrated the protective role of CED-13 against cisplatin-induced toxicity, although such a role might depend on the mtROS levels (García-Rodríguez et al., 2018). Our results suggest that *ced-13* also protects against the additive toxicity of cisplatin with PQ, pointing towards excessive cellular oxidation as the main consequence of such effect. The UPR^mt^ pathway activation under cisplatin exposure further suggests the impact of cisplatin on mtROS. Because imbalance of OXPHOS complexes is one of the leading causes of UPR^mt^ pathway activation (Choi et al., 2015), we studied *C. elegans* cellular respiration and quantified its reduction in the presence of cisplatin, glucose and PQ. Interestingly, we noticed that cisplatin exposure, and cisplatin in combination with glucose and PQ, negatively impacts cellular respiration at different levels.

Mitochondria play a crucial role in cisplatin cytotoxicity. In clinics, mitochondrial content has been proposed as a biomarker for platinum-based therapy response and as a target for cisplatin-resensitizing strategy (Cocetta et al., 2019). Mitochondrial accumulation of cisplatin triggers the imbalance of mitochondrial redox and signal crosstalk with the nucleus, altering cell metabolism (Choi et al., 2015). Moreover, mitochondrial dynamics are also relevant in cancer cells' adaptation mechanism to stressful conditions, including chemotherapy (Cocetta et al., 2019). The link between cisplatin cytotoxicity and mitochondrial dynamics has also been described in yeasts (Inapurapu et al., 2017). Consistently, our findings indicate that mitochondria play a crucial role in cisplatin cytotoxicity and support the use of *C. elegans* as a model to investigate cellular and systemic responses to cisplatin in the context of a pluricellular organism.

Based on our previous published cisplatin transcriptomic signature in *C. elegans*, we hypothesized that catecholamine-*O*-transferases genes might be involved in cisplatin-induced neurotoxicity. By CRISPR-Cas9, we generated deletion alleles for those COMT family members with higher transcriptional activity at postembryonic stages. These null alleles are viable and suitable for functional studies of nematode behavior and locomotion. Additionally, we generated a new deletion allele for tyrosine hydroxylase *cat-2*. Thus, we built a toolkit with DA-related mutants to investigate the impact of DA on cisplatin neurotoxicity. After evaluating several DA-dependent effects in the distinct mutant backgrounds, we noticed that *comt-3*, *comt-4* and *comt-5* exert differential effects on DA catabolism. Although *comt-5* has a major effect on body length, and *comt-4* and *comt-5* seem to affect animal locomotion, we did not find phenotypical alterations in the *comt-3* mutant. Functional redundancies among COMT genes in *C. elegans* should be explored in the future.

Recent publications have described the relevance of catecholamine metabolism in animal behavior (Omura et al., 2012; Rodríguez-Ramos et al., 2017). Here, we focused on animal locomotion, specifically path range, to evaluate the neuroprotective effect of DA in control and cisplatin conditions. First, we demonstrated that correct DA levels are essential to maintain normal levels of traveled distances, as evidenced in low- and high-DA mutants, *cat-2* and *comt-4* and *comt-5*, respectively, and when exogenous DA was administered. Interestingly, we found that exogenous DA also protects against cisplatin-induced animal locomotion and posture alterations, presumably caused by DA signaling perturbations because dopaminergic neuron integrity is not affected. Similar findings have been reported in a zebrafish model, in which high DA levels (or alternatively L-mimosine) not only protect against ototoxicity but also nephrotoxicity induced by cisplatin, without affecting the toxicity in tumoral cells (Wertman et al., 2020). Although further experiments are needed to unravel the mechanisms beyond such protectiveness, there are two hypotheses to explain why increased DA levels mediate oto- and nephroprotection. On the one hand, DA binding to D_1_ to D_5_ receptors, present in the kidney and mammalian inner ear, has been found to provide nephro- and cochlear nerve protection through increasing cAMP levels (Darrow et al., 2007; Gillies et al., 2015; Hans et al., 1990; Lendvai et al., 2011; Oestreicher et al., 1997; Ruel et al., 2001). Interestingly, D_1_, D_2_ and D_3_-like receptors are conserved in *C. elegans*. On the other hand, it has been proposed that DA could compete with cisplatin for organic cation transporters (OCTs), particularly for OCT2 (also known as SLC22A2), which is highly expressed in the kidney and the outer hair cells (Hucke and Ciarimboli, 2016). Indeed, treatment of patients with other cations, or disruption of OCT2 in mice, ameliorates cisplatin-induced toxicities (Hucke et al., 2019; Meijer et al., 1982; Zhang and Sulzer, 2012). Our findings provide additional pieces of evidence for the potential utility of DA to mitigate cisplatin-induced neurotoxic effects while avoiding the reduction of cisplatin doses.

## MATERIALS AND METHODS

### *C. elegans* strains and general methods

*C. elegans* strains were maintained using standard procedures (Stiernagle, 2006). Before conducting the experiments, animals were grown for at least two generations at the experimental temperature. Animals were synchronized using sodium hypochlorite (Porta-de-la-Riva et al., 2012). N2 was used as WT strain. Strains used in this study are listed in Table S2, including the ones generated by CRISPR-Cas9 and the ones provided by the Caenorhabditis Genetics Center (CGC). Mutants generated by CRISPR-Cas9 were outcrossed twice, and all the used strains were genotyped before use, using MyTaq™ DNA polymerase (Bioline) according to the manufacturer's instructions. Primers used for genotyping are listed in Table S3.

### CRISPR-Cas9 (Nested CRISPR)

Guide RNAs were designed using both Benchling (www.benchling.com) and CCTop (Stemmer et al., 2015) online tools. All CRISPR-Cas9 mutant and reporter strains were obtained following a co-CRISPR strategy (Kim et al., 2014), using *dpy-10* as a marker to enrich for genome-editing events (Arribere et al., 2014). In the case of CER588, *cat-2(cer181[cat-2p::gfp::h2b1-3])II*, Cas12a (Cpf1) was used instead of Cas9. For the last strategy, co-CRISPR was not viable because of the inefficient crRNA for *dpy-10*. In all cases, mixes were injected into gonads of young adult P_0_ hermaphrodites using a XenoWorks Microinjection System and following standard *C. elegans* microinjection techniques. F_1_ progeny were screened by PCR using specific primers, and F_2_ homozygotes were confirmed by Sanger sequencing. All the reagents for step 1 used in this study are listed in Tables S4 and S5. The injection mix conditions for Nested CRISPR step 1 and 2 are described in Vicencio et al. (2019), as well as universal sequences for step 2.

### Plates with special requirements

Plates with special requirements were prepared as follows. (1) Cisplatin plates: cisplatin (Accord) 1 mg/ml was used as a stock solution. For solid cisplatin plate preparation, 55 mm nematode growth medium (NGM) plates, with 10 ml agar, were prepared. The next day, 600 µl cisplatin solution stock was added on the surface to reach the desired concentration. When dried, 300 µl overnight OP50 cultures were seeded. (2) High-glucose plates: D-(+)-glucose powder (Sigma-Aldrich) was diluted in deionized water for stock solution preparation at the desired concentration; 300 µl from the respective stock solution was added to the plates before seeding and incubated overnight at room temperature. After incubation, plates were seeded with 300 µl overnight OP50 culture. (3) PQ plates: PQ (Sigma-Aldrich) powder was resuspended in dimethyl sulfoxide (DMSO) to reach 1 M as a stock solution. PQ solution was added to NGM (still melted), mixed and poured into 55 mm plates; 0.1 mM was used as final PQ concentration, and 300 µl overnight OP50 culture was seeded. (4) Low-peptone plates (tracking plates), for 1 l plates: 3 g sodium chloride, 20 g agar, 0.13 g bactopeptone and 1 l desionized water were mixed and autoclaved. Then, 3.5 cm plates were prepared with this solution plus standard concentration buffers (Stiernagle, 2006). Plates were seeded with a single drop in the middle of the surface from an overnight OP50 culture the day before the experiment.

### Body length assay

A synchronized population of L1-arrested larvae was cultured on NGM plates containing fresh OP50 and 60 µg/ml cisplatin at 20°C. The body lengths of 50 animals for each condition were measured at 72 h on a stereomicroscope using NIS-Elements 3.2 imaging system. Each assay was done in triplicate, and two biological replicates were performed for each condition.

### *hsp-6::GFP* quantification

For quantification of *hsp-6::GFP* expression, animals, exposed or not to 100 µg/ml cisplatin from L4/YA stage for 24 h, were transferred to a microscope slide and anesthetized with 15 µl of 10 mM levamisol hydrochloride (Sigma-Aldrich, 31742) dissolved in S-Basal [5.85 g NaCl, 1 g K_2_HPO_4_, 6 g KH_2_PO_4_, 1 ml cholesterol (5 mg/ml in ethanol), H_2_O to 1 l], and sealed with a cover slip. Worms were pictured at 25× magnification using an Imager2 Zeiss fluorescence microscope, and the same exposure time was applied to all experimental conditions. GFP expression was quantified using ImageJ software (https://imagej.nih.gov/ij/), measuring the fluorescence intensity of the pharyngeal bulb region of each worm acquired. Three experimental replicates were performed, and, for each condition, 10-15 worms were pictured and quantified.

### *gst-4::GFP* quantification

Synchronized animals were treated with 60 µg/ml cisplatin for 24 h. Then, levamisol hydrochloride (Sigma-Aldrich, 31742)-anesthetized animals were mounted on a microscope slide and covered with a cover slip. Worms were pictured using an Imager2 Zeiss fluorescence microscope, and the same exposure time was applied to all experimental conditions. GFP expression was quantified using ImageJ software, measuring the fluorescence intensity of the whole animal. Three experimental replicates were performed. For each condition, at least 20 worms were acquired and quantified.

### OCR assessment

*C. elegans* respirometry profile was determined by measuring OCR by a Seahorse XFe96 Analyzer (Agilent). The optimized procedure proposed in Koopman et al. (2016) was followed with minor modifications. OCR was calculated in N2-treated animals under six different conditions: 60 µg/ml cisplatin, 0.1 mM PQ, 40 µM glucose, PQ+cisplatin, glucose+cisplatin and H_2_O as a vehicle. The protocol described in Koopman et al. (2001) was followed with minor modifications. Plates with special requirements were freshly prepared as indicated above. We ensured that animals were well synchronized and, on the day of the experiment, did not exceed the L3 stage because after the L3/L4 molt substantial differences in mitochondrial load would exist between younger and older animals, affecting respirometry (Bratic et al., 2009; Tsang and Lemire, 2002). This experiment was performed three independent times. For each condition, eight biological replicates (20 animals per replicate) were analyzed.

### Automated tracking of behavioral features

Synchronized animals were grown at 20°C on NGM plates from L1 for 34 h. Then, animals were plated on 12-well plates (30 animals per well) containing liquid culture for each condition: control, cisplatin (Accord, 1 mg/ml) or DA (Sigma-Aldrich) at the desired concentration for 24 h treatment. As a source of food, 25 ml overnight OP50 culture diluted in 5 ml M9 was used. Three biological replicates were prepared for each condition. The day of the experiment, animals were recovered, washed in M9, and finally seeded on tracking plates and allowed to habituate for 10 min. Animals were recorded for 15 min using Tierpsy Tracker, and data were extracted and analyzed using Tierpsy Tracker 2.0 software (Javer et al., 2018a,b).

### Dopaminergic neuron integrity evaluation

Synchronized L4/YA animals were exposed to 250 µg/ml cisplatin for 24 h. Dopaminergic neurons were identified and imaged by detection of *dat-1p::GFP* signal using a Leica TCS SP5 confocal laser scanning microscope. Imaging was done at 63× magnification in *Z*-stacks. Thirty animals per condition were evaluated. We used Zeiss Zen 2012 (Blue Edition), FIJI (ImageJ version 2.0.0-rc-68/1.52p) for image processing.

### Graph plotting and statistical analysis

Data were plotted and statistical analyses were conducted using GraphPad Prism 8.0. *P*<0.1 was considered statistically significant.

## Supplementary Material

Supplementary information
