# Supplementary material for: Insights into cisplatin-induced neurotoxicity and mitochondrial dysfunction in Caenorhabditis elegans
Source: Dis Model Mech. 2022 Mar 31;15(3):dmm049161. doi: 10.1242/dmm.049161 (PMC8995082; doi:10.1242/dmm.049161)
Supplement: Supplementary information [file dmm-15-049161-s1.pdf]

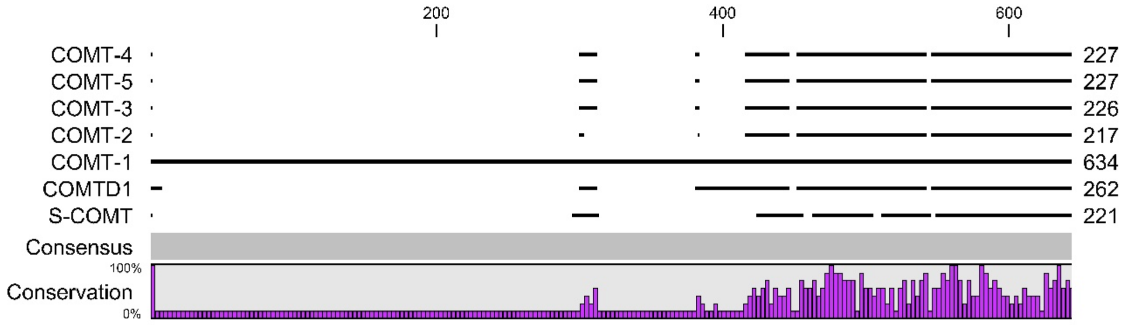

Fig. S1. Phylogenetic analysis of *C. elegans* COMT members. Scheme of COMT-1 to -5, COMTD1 and S-COMT protein sequences (black lines) and conserved residues (purple bars). Alignments were illustrated with CLC Sequence Viewer 8.0.

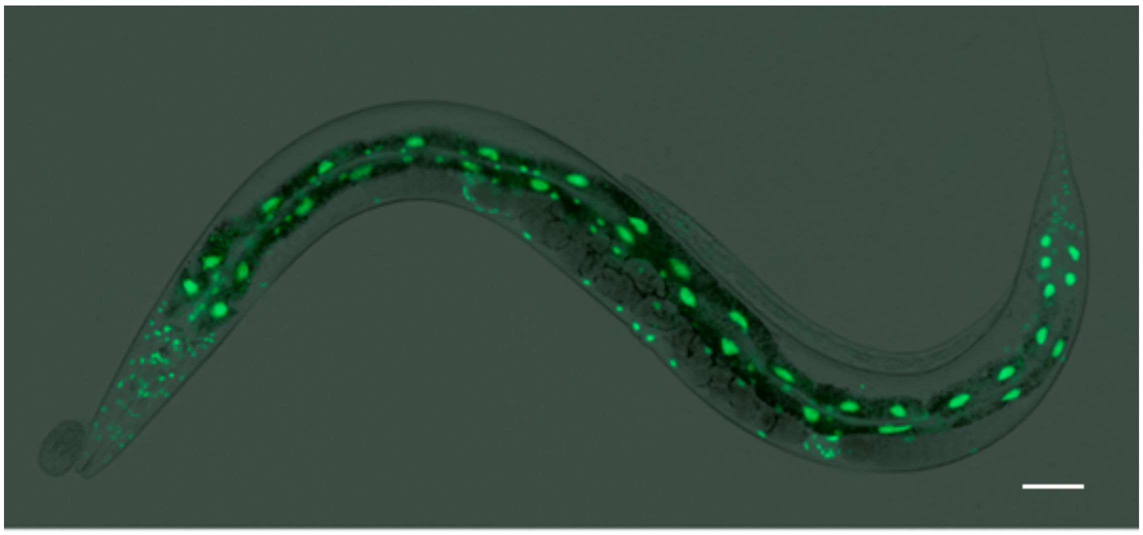

Fig. S2. COMT-4 is expressed in neuronal cells. *comt- 4(cer157[comt-4p::GFP::H2B])* nuclear signal in an adult animal. Scale bar means 50 µm.

Disease Models & Mechanisms • Supplementary information

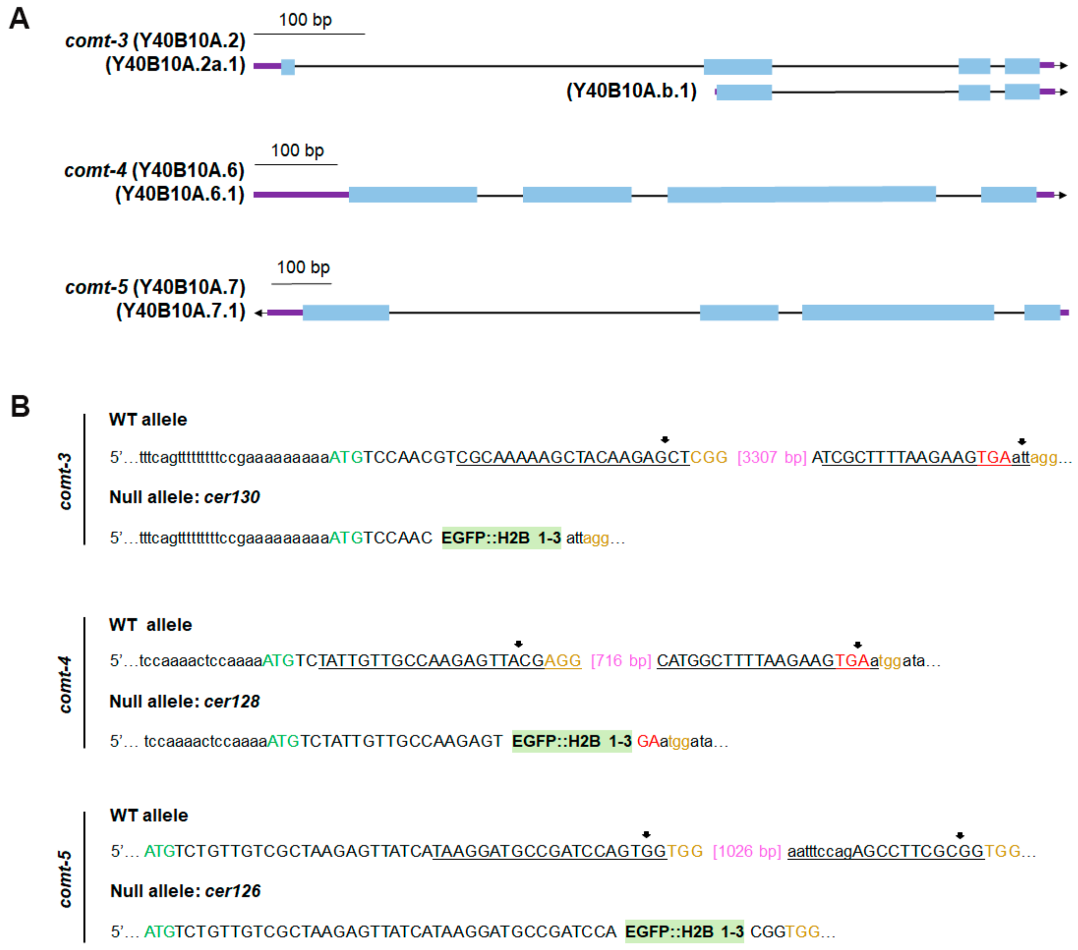

Fig. S3. *comt-3*, *comt-4* and *comt-5* loci and null allele molecular designs. A. Schematic representation of *comt-3*, *comt-4* and *comt-5* transcripts. Introns are shown by black lines, 5´and 3´UTRs (untranslated regions) by purple bars and exons by light blue bars. **B.** Illustration of the sense strand sequences before and after homology-directed repair by CRISPR-Cas9 producing *cer130, cer128* and *cer126* null alleles. Start and stop codons are represented in green and red letters, respectively. PAM sequences are shown in yellow. 3´and 5´crRNA sequences are underlined and cut sites are shown by black arrows. The resulting deletion lengths are represented in pink and EGFP::H2B fragments 1 and 3 are represented by green shadow. Partial 5' and 3' UTR sequences are indicated in lowercase.

Disease Models & Mechanisms • Supplementary information

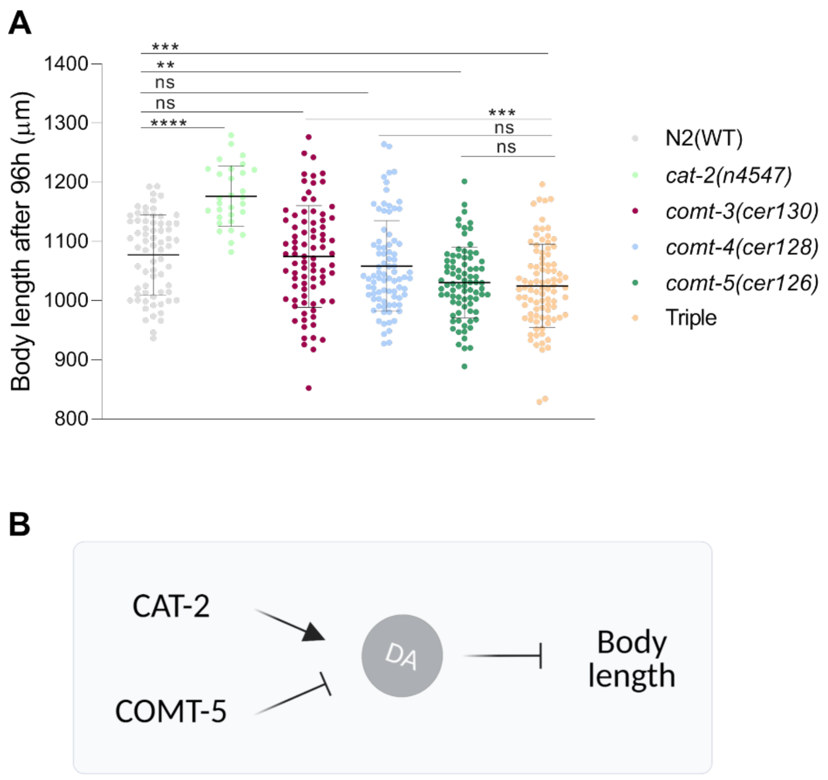

Fig. S4. Body length characterization of the dopamine signaling-related mutants. Dots represent measurement of individual animals from three independent experiments, and black lines depict median and interquartile range. Strains used in these experiments are simple deletion alleles for *comt-3*, *comt-4*, *comt-5*, triple mutant for those, and deletion allele for *cat-2*. The body length of fifty animals for each condition were analyzed in each experiment. Statistical significances was assessed by one-way ANOVA (Krustal-Wallis and Dunn's tests). **, ***, **** and ns mean p<0.01, p<0.001, p<0.0001 and no significant, respectively.

Disease Models & Mechanisms • Supplementary information

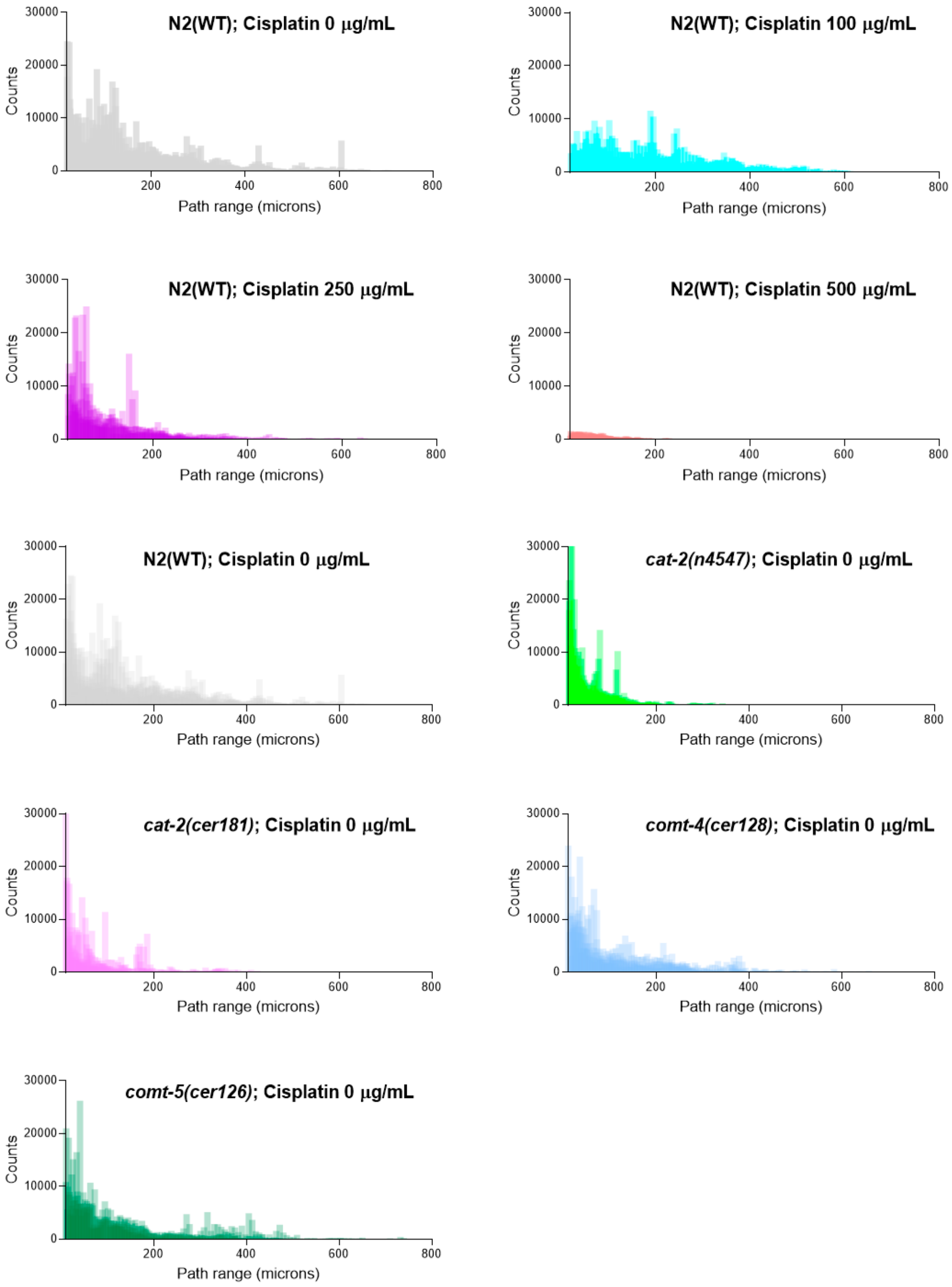

Fig. S5. Path range analysis of wild type, *cat-2*, *comt-4* and *comt- 5* mutants. Histograms show the path range profile of each genotype in control or cisplatin conditions. The experiment was performed twice and 30 animals per conditions were analyzed in each replicate.

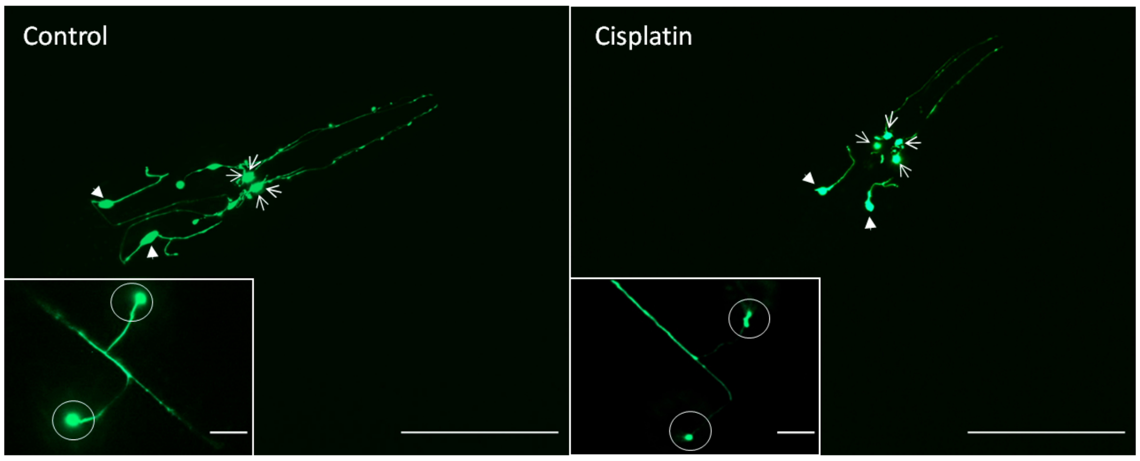

Fig. S6. Cisplatin does not affect dopaminergic neurons integrity. Representative images of *dat-1p::GFP* animals showing four CEP and two ADE neurons in the head (big panels, scale bars mean 100 μm) and two PDE neurons in the posterior half of the body (small panels, scale bars mean 10 μm).

Disease Models & Mechanisms • Supplementary information

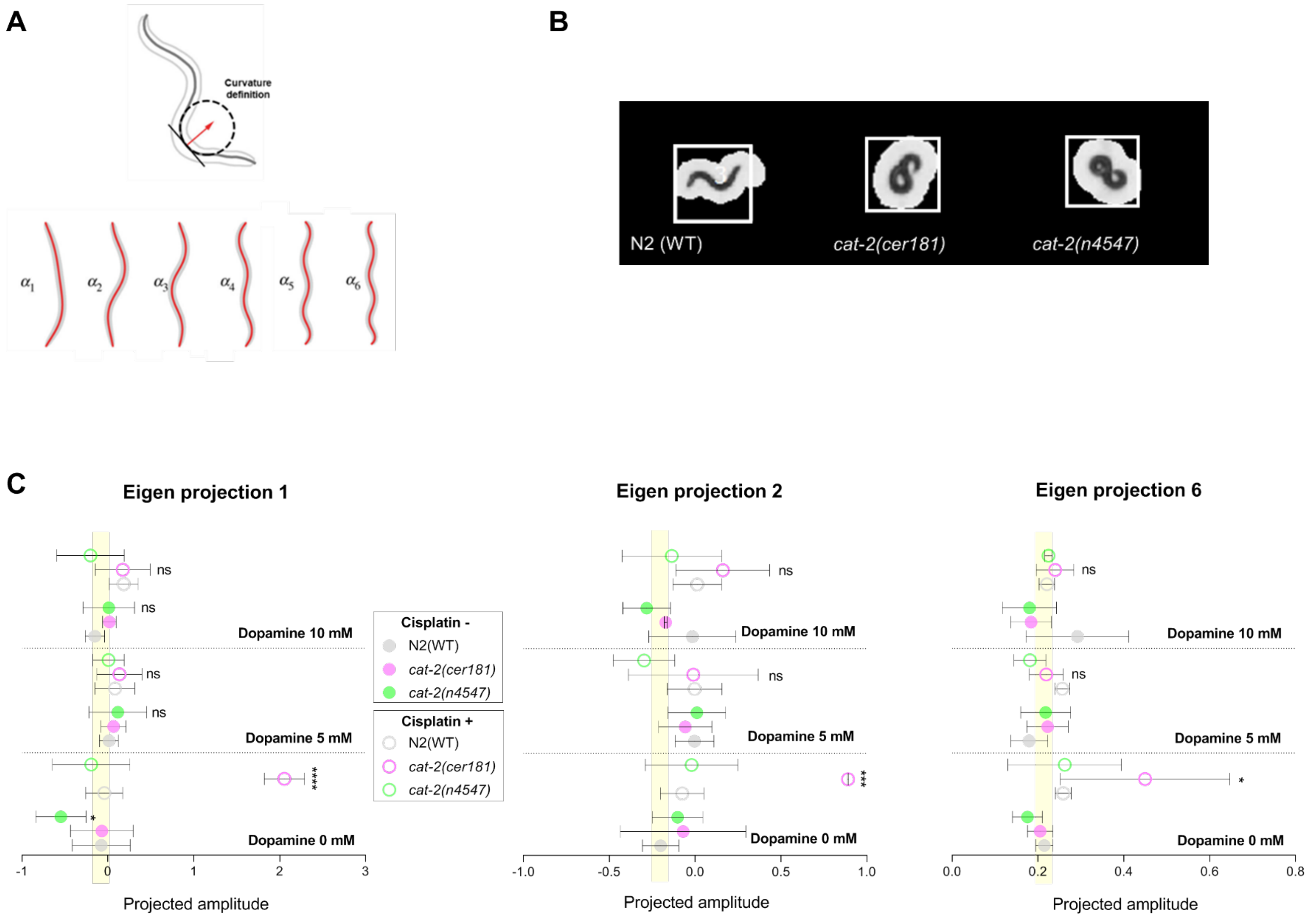

Fig. S7. Body posture alteration of dopamine defective mutants induced by cisplatin. A. On top of the figure, a worm diagram is illustrated, crossed by a line describing the shape. Curvature definition is given by the arc length function, represented by the red arrow. On the bottom, schematic representation of six eigenprojections accounting for almost the entire variance in body shape (Modified from Javer et al., 2018b). **B.** Images of animals exposed to cisplatin showing body posture alterations visualized by Tierpsy Tracker 2.0. **C.** Graph show projected amplitudes α_1, α_2 and α_6 of dopamine-defective mutants. Circles represent the mean of two independent experiments and bars represent standard deviation. Yellow shadows point out the projected amplitude for wild-type animals in the absence of cisplatin nor dopamine. *, **, ns mean p<0.1, p<0.01 and not significant, respectively. Statistics were analyzed by one-way ANOVA (Krustal-Wallis and Dunn's tests).

Disease Models & Mechanisms • Supplementary information

Table S1. BLASTP analysis of *C. elegans* COMT proteins compared to human COMDT1 and S-COMT.

Query sequence	Protein	Query cover (%)	Identity (%)
COMTD1	COMT-4	63	46.15
	COMT-3	63	41.42
	COMT-2	63	43.53
	COMT-5	63	43.20
	COMT-1	67	32.43
S-COMT	COMT-4	77	36.9
	COMT-3	17	74.47
	COMT-2	17	76.60
	COMT-5	17	65.96
	COMT-1	63	22.45

Table S2. Strains used in this study.

Strain	Genotype	Reference
CER494	*comt-5(cer126 [comt-5p::gfp::h2b 1-3]) V*	This study
CER496	*comt-4(cer128[comt-4p::gfp::h2b 1-3]) V*	This study
CER498	*comt-3 (cer130[comt-3p::gfp::h2b 1-3) V*	This study
CER587	*comt-3 (cer166[comt-3p::gfp::h2b 1-3); comt-4(cer128 [comt-4p::gfp::h2b 1-3]; comt-5(cer167 [comt-5p::gfp::h2b 1-3]) V*	This study
CER497	*comt-4(cer128 [comt-4p::gfp::h2b 1-3] comt-3 (cer166[comt-3p::gfp::h2b 1-3) V*	This study
CER554	*comt-4(cer157[comt-4p::GFP::H2B]) V*	This study
CER588	*cat-2(cer181 [cat-2p::gfp::h2b 1-3]) II*	This study
MT15620	*cat-2(n4547) II*	CGC
FX536	*ced-13(tm536) X*	CGC
MD792	*ced-13(sv32)*	CGC
SJ4100	*zcIs13[hsp-6::GFP] V*	CGC
BY250	*vtIs7[pRB490 (Pdat-1::GFP)]V*	Kindly provided by Dr Antonio miranda
CL2166	*dvIs19 [pAF15(gst-4p::GFP::NLS)] II*	CGC

Disease Models & Mechanisms • Supplementary information

Table S3. List of primers used for genotyping.

Gene	Allele		Primer Fwd	Primer Rev
comt-4	*cer128* *cer157*		TCCAAAGTTCAGTTCGGAAG	CCAGAAATCGGACTTGATTGA
comt-3	*cer130* *cer166*		cttgttctctcggactatctgatag	TGTCTACTTTGCCCCCAATG
comt-5	*cer126* *cer167*		ccctccaaacagctattgaaacg	ACATGAGTTCCATCGCCAAGA
cat-2	*n4547* *cer181*	WT	ctatgtgaagtcacacctgtc	gagatcacggatcacaagag
	cer181	Mut		cttgctggaagtgtacttggtg
ced-13	*tm536*		GTCAGGTGGCCCACGAAAC	CAGTACGTGCTTGATGCAC
	sv32			GGCAGTTGCTGAGACGTTG

Table S4. List of crRNAs used for Nested CRISPR step 1.

Name	Generated allele	Sequence
comt-4 5'	*cer128*	TATTGTTGCCAAGAGTTACG
comt-4 3'		TTCACTTCTTAAAAGCCATG
comt-3 5'	*cer130, cer160*	CGCAAAAAGCTACAAGAGCT
comt-3 3'		TCGCTTTTAAGAAGTGAATT
comt-5 5'	*cer126, cer167*	TAAGGATGCCGATCCAGTGG
comt-5 3'		AATTTCCAGAGCCTTCGCGG
cat-2 5'	*cer181*	CGTGATCCTCTCCAGAGCCC
cat-2 3'		ACATTGTAATCGATATTTTC

Table S5. List of ssODN used for Nested CRISPR step 1.

Locus	Generated allele	Sequence
comt-4	*cer128, cer157*	TTTCAGTTTTTTTTTTCCGAAAAAAAAAAATGTCCAACccaagtttgtacaaaaaagcaggc tccatgagtaaaggagaagaacttttcactggagagggaaccaaggccgtcaccaagtacacttccagcaag taaATTAGGGGCTTTTTTTTTAATTTTGAATTATATTTA
comt-3	*cer130, cer166*	tttcagttttttttttccgaaaaaaaaaaATGTCCAACccaagtttgtacaaaaaagcaggctccatgagtaaag gagaagaacttttcactggagagggaaccaaggccgtcaccaagtacacttccagcaagtaaattaggggctt tttttttaattttgaattatattta
comt-5	*cer126, cer167*	GTTGTCGCTAAGAGTTATCATAAGGATGCCGATCCAccaagtttgtacaaaaaagcag gctccatgagtaaaggagaagaacttttcactggagagggaaccaaggccgtcaccaagtacacttccagca agtaaCGGTGGCTCCGTAGCTGACGAGAAAAGACGAGAAGA
cat-2	*cer181*	TCGTTGTTGGCGTGGGGACCCCTTGAAAAATTGGAAGAGGAAATGTTTTTG CGGTATATGCGGAGGCGGGGCagtaaaggagaagaacttttcactggagttgtcccaattgccgt gtctgagggaaccaaggccgtcaccaagtacacttccagcaagTGAAACCTAATTTACCTAATA CTTTGCTAAACTAT

Disease Models & Mechanisms • Supplementary information

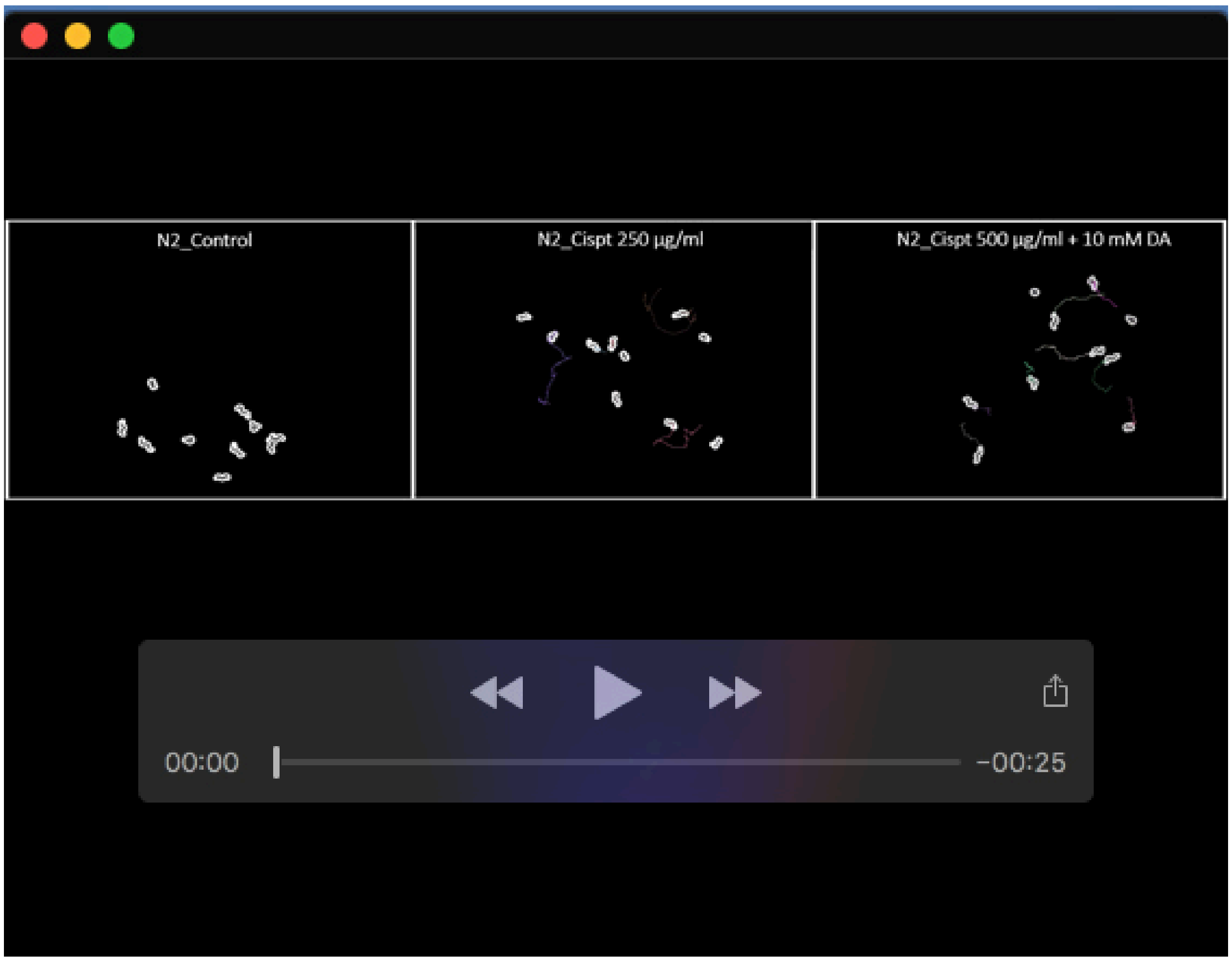

Movie 1. Cisplatin effect on animals' path range 1. Representative videos showing wild-type animals moving at control, 250 µg/mL and 500 µg/mL cisplatin conditions. Colored lines represent the path range of individual animals.

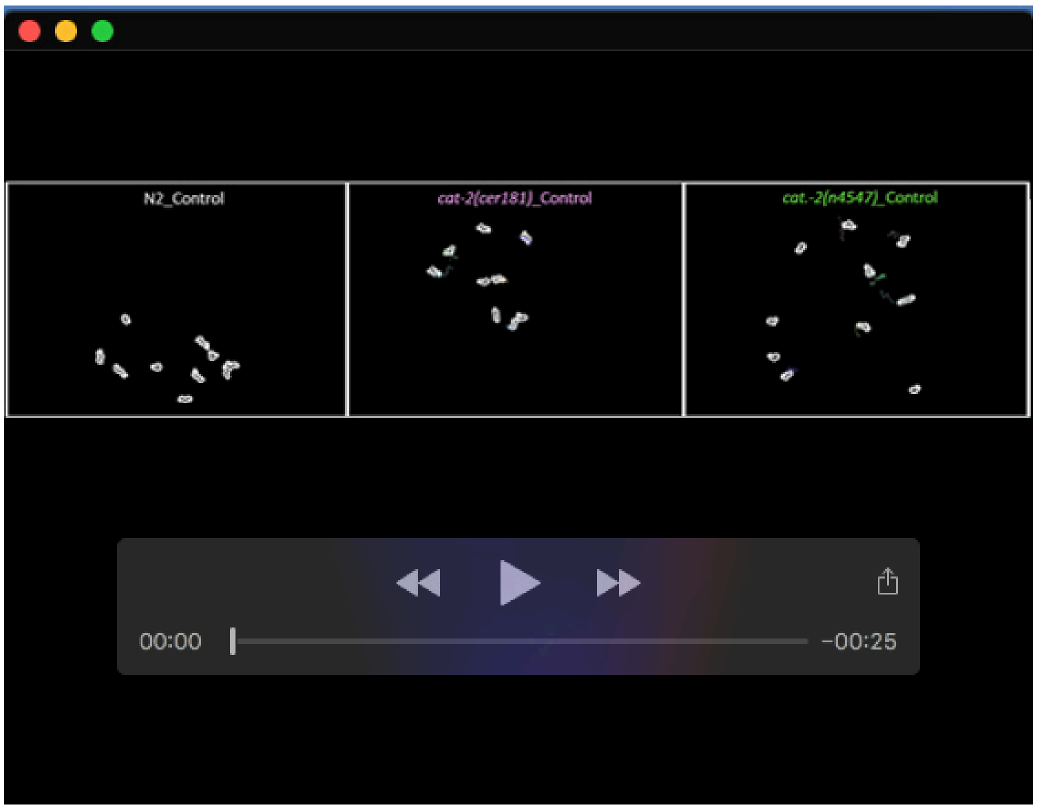

Movie 2. Reduced path range of low dopamine mutants. Representative videos showing wild-type and *cat-2* mutants (*n4547* and *cer181*) moving at control condition. Colored lines represent the path range of individual animals.

Disease Models & Mechanisms • Supplementary information

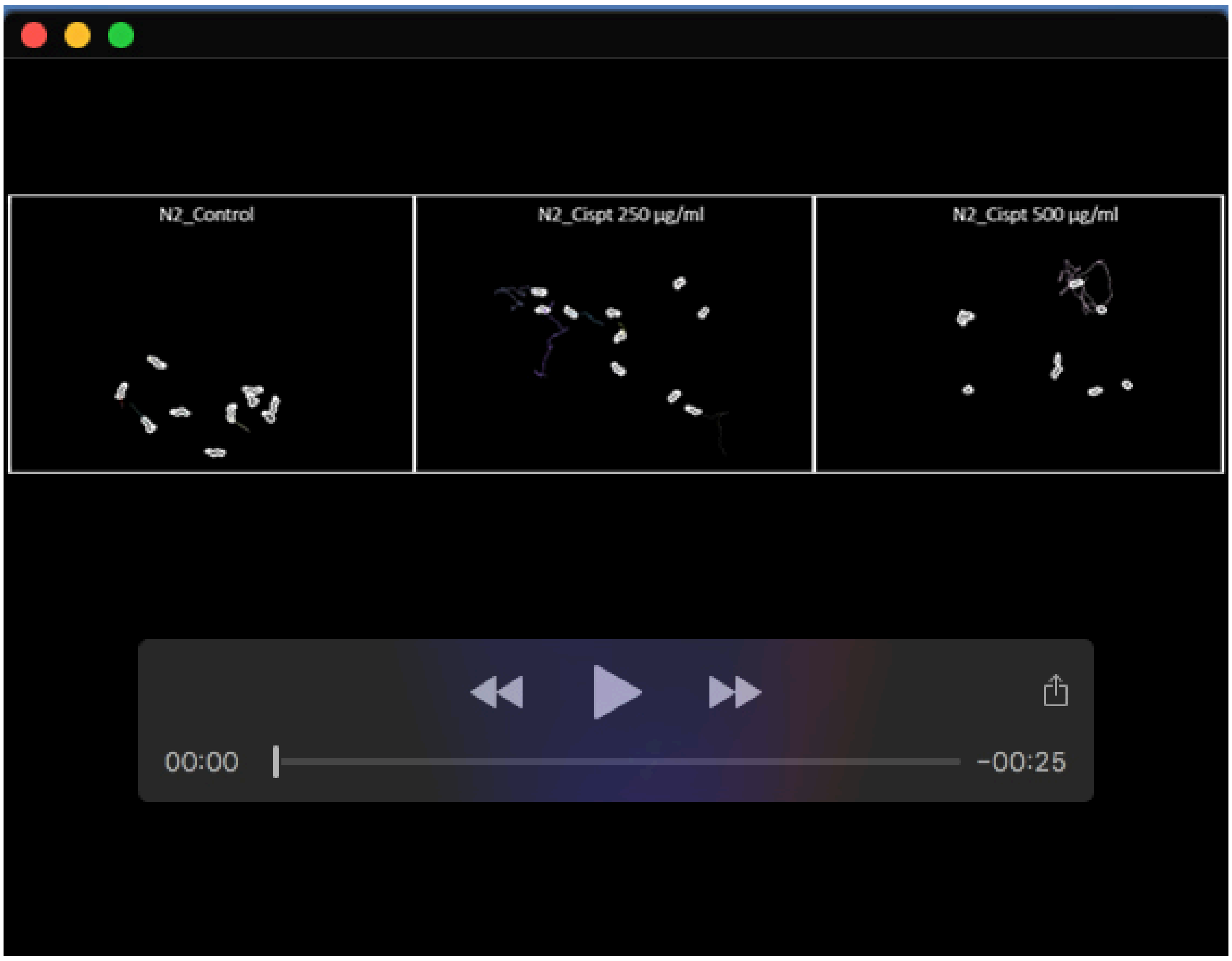

Movie 3. Dopamine rescues cisplatin-induced aberrant path range. Representative videos showing wild-type animals moving at control, 250 µg/mL cisplatin and 250 µg/mL cisplatin + 10 mM DA. Colored lines represent the path range of individual animals.
